# Substitutional synthesis of sub-nanometer InGaN/GaN quantum wells with high indium content

**DOI:** 10.1038/s41598-021-99989-0

**Published:** 2021-10-18

**Authors:** I. G. Vasileiadis, L. Lymperakis, A. Adikimenakis, A. Gkotinakos, V. Devulapalli, C. H. Liebscher, M. Androulidaki, R. Hübner, Th. Karakostas, A. Georgakilas, Ph. Komninou, E. Dimakis, G. P. Dimitrakopulos

**Affiliations:** 1 Department of Physics, Aristotle University of Thessaloniki, Thessaloniki, Greece; 2 Max-Planck Institut für Eisenforschung GmbH, Düsseldorf, Germany; 3 Microelectronics Research Group (MRG), IESL, FORTH, Heraklion, Greece; 4 Department of Physics, University of Crete, Heraklion, Greece; 5 Institute of Ion Beam Physics and Materials Research, Helmholtz-Zentrum Dresden-Rossendorf, Dresden, Germany

**Keywords:** Engineering, Materials science, Nanoscience and technology, Physics

## Abstract

InGaN/GaN quantum wells (QWs) with sub-nanometer thickness can be employed in short-period superlattices for bandgap engineering of efficient optoelectronic devices, as well as for exploiting topological insulator behavior in III-nitride semiconductors. However, it had been argued that the highest indium content in such ultra-thin QWs is kinetically limited to a maximum of 33%, narrowing down the potential range of applications. Here, it is demonstrated that quasi two-dimensional (quasi-2D) QWs with thickness of one atomic monolayer can be deposited with indium contents far exceeding this limit, under certain growth conditions. Multi-QW heterostructures were grown by plasma-assisted molecular beam epitaxy, and their composition and strain were determined with monolayer-scale spatial resolution using quantitative scanning transmission electron microscopy in combination with atomistic calculations. Key findings such as the self-limited QW thickness and the non-monotonic dependence of the QW composition on the growth temperature under metal-rich growth conditions suggest the existence of a substitutional synthesis mechanism, involving the exchange between indium and gallium atoms at surface sites. The highest indium content in this work approached 50%, in agreement with photoluminescence measurements, surpassing by far the previously regarded compositional limit. The proposed synthesis mechanism can guide growth efforts towards binary InN/GaN quasi-2D QWs.

## Introduction

III-nitride semiconductors have led to breakthroughs in optoelectronic devices due to their large range of direct band gaps, high carrier mobilities, and capacity for alloying. In_*x*_Ga_1−*x*_N/GaN quantum well (QW) heterostructures are key elements of active regions in devices, such as lasers and light emitting diodes^1,2^. A current bottleneck towards highly-efficient chromatically-tunable devices operating across the optical spectrum, relates to the possibility of bandgap engineering by tuning the indium content. In particular, the reduction of the internal quantum efficiency at high indium contents is attributed in part to the high misfit to GaN that promotes plastic relaxation, thus reducing carrier recombination efficiency^3–5^. Moreover, the high elastic strain in pseudomorphic QWs causes carrier separation through the quantum-confined Stark effect (QCSE)^6^.

Short period superlattice (SL) nanostructures comprising ultra-thin QWs have been proposed to mitigate these problems. Such QWs have monolayer (ML) scale thickness and can form digital alloys with GaN when arranged in short-period multi-QWs (MQWs). The ultra-thin QWs can sustain large elastic strains without plastic relaxation, and reduce the QCSE. Theoretical investigations have shown that the strain and bandgap of In_*x*_Ga_1−*x*_N/GaN SLs can be adjusted through the QW and barrier thicknesses^7–11^. In addition to providing bandgap tunability, MQW SLs allow for strain transfer to the barriers, and may reduce the compositional fluctuations and indium clustering that significantly affect the bandgap^12,13^. Moreover, a theoretically proposed and experimentally indicated possibility to achieve topological insulator behavior using In_*x*_Ga_1−*x*_N/GaN SLs with high indium content could revolutionize III-nitride applications by opening up new fields in spintronics and quantum computing^14–16^.

Increasing the indium content of In_*x*_Ga_1−*x*_N/GaN MQW SLs has been the target of growth efforts, but such attempts have not been very successful so far due to the high lattice mismatch (up to 11% between InN and GaN, and ≈1 ML critical thickness), and the much lower deposition temperature (*T*_d_) of InN relative to GaN. Plasma-assisted molecular beam epitaxy (PAMBE) was proposed by Yoshikawa et al*.*^17–19^ to grow 1 or 2 ML-thick InN QWs, using *T*_d_ up to 650 °C i.e., much higher than the decomposition temperature of InN i.e., *T*_dec_ ≈ 500 °C. It was claimed that a dynamically mobile In/N adlayer can be ‘frozen’ in place through fast capping by the GaN barriers to avoid selective nitrogen desorption. This stabilization was attributed to the nitrogen atoms directly below the indium atoms being strongly bonded to gallium atoms.

However, the assertion that binary InN/GaN QWs can be deposited was disputed by other authors claiming that the indium content is kinetically limited to a maximum of *x*_In_ ≈ 25–33%^20–22^. Such studies employed almost stoichiometric or N-rich growth conditions and were correlated to In_*x*_Ga_1−*x*_N alloy ordering^23^. A recent study comprising metal-rich conditions showed an incommensurate indium adlayer, indicating that kinetically stabilized ordered surface reconstructions were not imposed in this case^24^. Still, the presented results showed formation of 1 or 2 ML In_*x*_Ga_1−*x*_N QWs. In previous work, we determined directly the indium content of QWs grown at 680 °C using quantitative high-resolution scanning transmission electron microscopy (HRSTEM) observations, and again yielded *x*_In_ < 35%^25^. Photoluminescence (PL) measurements performed on such SLs revealed emission peaks ranging between 2.8 and 3.4 eV^17,21,24,26–29^, when first principle calculations predict ≈2.1 eV for 1InN/*n*GaN SLs in the limit of a large number, *n*, of GaN barrier MLs^7,30^. This deviation has been attributed either to formation of In_*x*_Ga_1−*x*_N alloy or to reduced spatial localization of excitons^17,30^.

According to theoretical calculations, In_*x*_Ga_1−*x*_N alloy orderings can be epitaxially stabilized for local strain accommodation^31^. The $(\sqrt{3}\times \sqrt{3})$ R30° surface reconstruction allows stabilization of ML-thick QWs with *x*_In_ = 33%, while there exists an upper limit of *x*_In_ = 25% with the $(2\sqrt{3}\times 2\sqrt{3})$ R30° configuration under N-rich growth conditions^23^. However, there is much less understanding of what takes place under metal-rich conditions. An In-Ga exchange mechanism has been proposed to take place in the indium adlayer during capping with the GaN barriers^24^.

Overall, the deposition mechanisms of In_*x*_Ga_1−*x*_N/GaN QWs remain unclear regarding the stabilization of the QWs, the processes in the adlayer, and the coupling between composition and strain. A compositional limit seems to be supported by previous results. In this contribution, we carry out a systematic study of In_*x*_Ga_1−*x*_N/GaN MQWs grown by PAMBE under metal-rich conditions at *T*_d_ between 470 and 640 °C, aiming to elucidate indium incorporation and suggest a deposition model that allows to identify growth routes which kinetically stabilize much higher indium contents. HRSTEM provides a unique way for direct determination of the composition of ultra-thin QWs through Z-contrast quantification^25^. In this work the indium content is determined with unparalleled spatial resolution by performing probe-corrected HRSTEM observations and extensive multislice simulations in order to compare experimental images to energetically relaxed atomic supercells. This analysis is combined with ML-scale strain measurements to yield the strain-composition coupling. The results are correlated to the bandgap of the heterostructures using PL spectroscopy. The thermodynamics of indium incorporation are investigated using density functional theory (DFT) calculations.

## Results

### Sample structures

**Figure 1:**
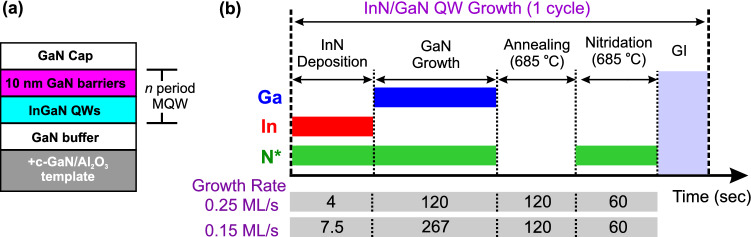
(**a**) Schematic of the sample structure for *n* = 5 (sample series A) or *n* = 3 (sample series B) MQWs. (**b**) Deposition sequence for the two employed growth rates. The duration of the growth interruption (GI) step was ≈8–9 min depending on temperature.

Figure 1a illustrates schematically the grown MQW heterostructures. The metal-rich growth conditions are summarized in Table 1, and Fig. 1b illustrates schematically the employed growth regime. We distinguish two groups of samples. Series A consists of samples with QW deposition temperatures *T*_d_ in the range of 550–640 °C, whereas the growth temperature *T*_b_ of the GaN barriers was the same as that of the QWs. In series B, the QWs as well as the first 1 nm of the barriers were deposited at *T*_d_ = 470 °C, while the remaining 9 nm of the barriers were grown at a higher temperature (*T*_b_ = 550–650 °C) in order to obtain high crystal quality. The MQWs of series A comprised five periods, and those of series B three. Each growth cycle comprised stages of high temperature annealing and nitridation in order to fully remove any metal adatoms prior to deposition of the QW.

**Table 1:** List of samples with QW and barrier deposition temperatures *T*_d_ and *T*_b_, respectively, flux ratios (*F*_In_/*F*_N*_), growth rates (*F*_N*_), observed QW thickness (*L*_QW_) and indium contents *x*_In_ derived from the strain and Z-contrast measurements. In sample series B, the first 1 nm of the barriers was grown at 470 °C.

Sample	*T*_d_ (^o^C)	*T*_b_ (^o^C)	*F*_In_:*F*_N*_	*F*_N*_ (ML/s)	*L*_QW_ (ML)	Indium content *x*_In_ (at%)	
						By strain	By Z-contrast
A1	640	640	3	0.25	1	28 ± 3	29 ± 4
A2	600	600	3	0.25	1	35 ± 4	30 ± 4
A3	550	550	3	0.25	1	41 ± 4	47 ± 4
A4	550	550	6	0.15	1	44 ± 4	48 ± 3
B1	470	600	3	0.25	2	29 ± 3	27 ± 2
B2	470	550–650^a^	3	0.25	2	29 ± 2	29 ± 3
B3	470	550	6	0.15	2	28 ± 3	28 ± 1

^a^Temperature gradient.

**Figure 2:**
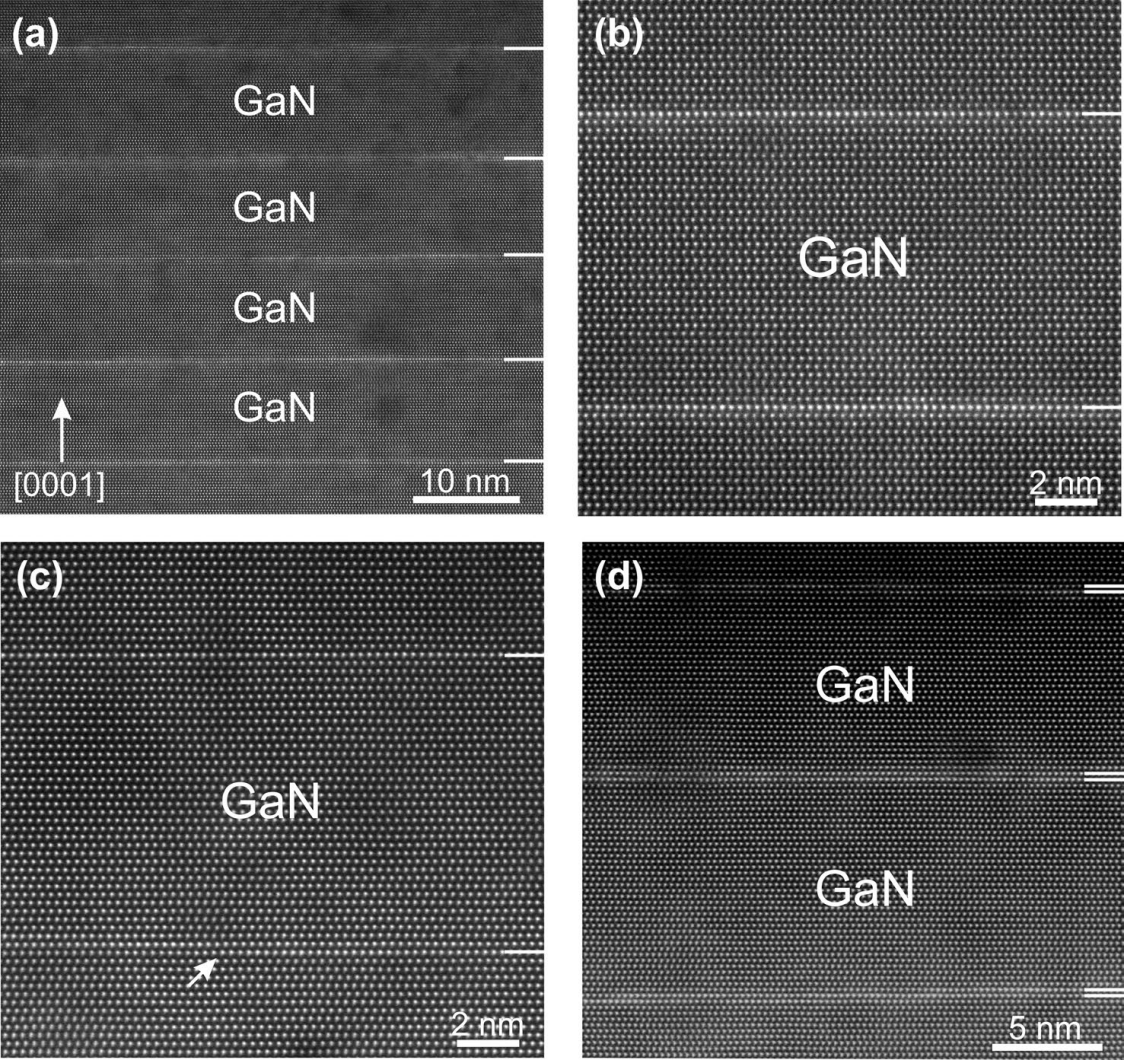
Cross-sectional HRSTEM images of the MQW heterostructures recorded along the [${\overline{1}}{\overline{1}}20$ ] zone axis. (**a**) Representative overall image obtained from sample A3 showing the good periodicity of the MQW. (**b–d**) Atomic resolution HAADF-HRSTEM images of samples A3, A4, B3 respectively. In series A, 1 ML-thick QWs are observed and, in series B, 2 ML-thick QWs are formed. The tilted arrow in (**c**) indicates a step of the QW.

Transmission electron microscopy (TEM) observations showed an excellent overall crystal quality for all samples in the A series. Figure 2a illustrates a representative lower magnification high-angle annular dark field (HAADF) HRSTEM image of sample A3 showing the good MQW periodicity. In the A sample series, the average thickness of the barriers was 38 ± 1 MLs, and in the B sample series 32 ± 2 MLs. TEM diffraction contrast observations revealed that sample A3 exhibited few basal stacking faults (SFs) due to the relatively lower deposition temperature. SFs were not observed in sample A4, albeit growth at the same temperature, probably due to the lower growth rate. Figure 2b,c present atomic resolution Z-contrast HAADF-HRSTEM images from the A sample series, whereby it is seen that in all cases 1 ML QWs are obtained, with excellent crystal structure. The QWs exhibited few steps of ML height; other than that, they were very well confined in 1 ML. In sample A4, the In/N* flux ratio was doubled by lowering the active nitrogen (N*) flux to about a half.

The possibility of alloy ordering in our QWs was checked by aberration-corrected high resolution TEM (HRTEM) observations along the [$1 {\overline{1}}00$] zone axis in thinner foil areas, as required to show this phenomenon^23^. We did not obtain experimental indications of ordering in our samples except for the high temperature sample A1 which exhibited few sparse patches (≈1 nm in length) but not a continuous ordered structure.

Interestingly, for *T*_d_ < 550 °C, the QWs started to exhibit a thickness of ≈2 MLs. However, when the same temperature was used for the barriers (i.e., *T*_d_ = *T*_b_), the crystal quality deteriorated by the overlap of many SFs, resulting in a mixture of wurtzite and zincblende structure. This problem was successfully resolved by employing the aforementioned increase of *T*_b_ after the first 1 nm GaN in sample series B. In sample B3, the fluxes and growth rate were similar to A4. In all cases, high quality MQWs with no newly introduced SFs or threading dislocations was achieved (see Supplementary Figs. S1 and S2 online). In Supplementary Fig. S2 online, MQWs grown at *T*_d_ = 470 °C with and without temperature ramping in the barriers are compared. Figure 2d shows a representative atomic resolution HAADF-HRSTEM image of sample B3 comprising three QWs with 2 ML thickness on average. However, the Z-contrast was not homogeneous along the QWs, and they exhibited a more corrugated structure. We also compared the series B samples to MQWs grown at 470 °C without increasing the barrier growth temperature. In the latter, the first QW is not affected by the defects introduced above it. It was thus established that, in sample series B, the QW composition and thickness were not affected by the increased *T*_b_ (see Supplementary Fig. S3 online) which is attributed to the protective 1 nm GaN grown at 470 °C.

### Strain characterization and strain-composition coupling in ultra-thin InGaN QWs

**Figure 3:**
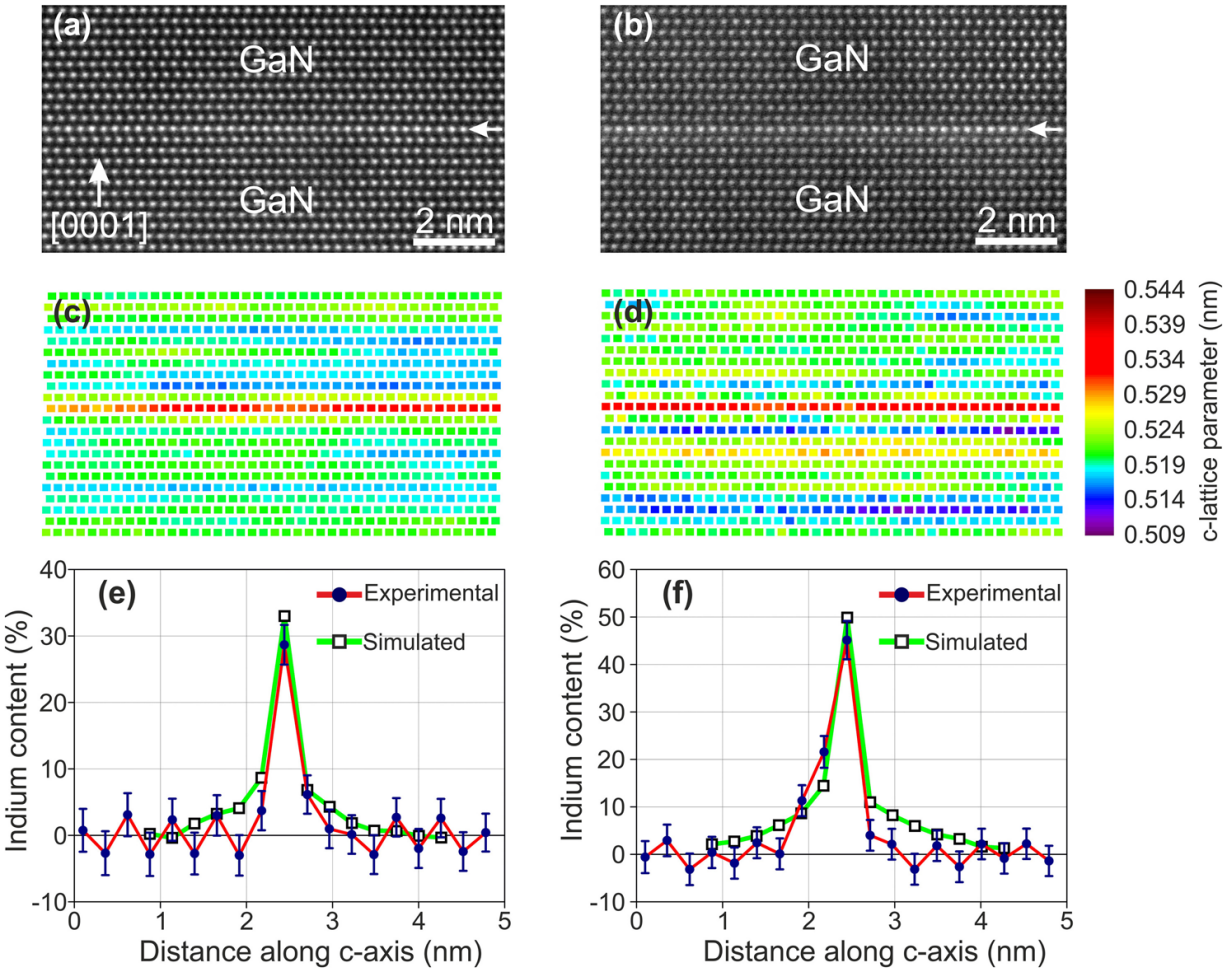
Representative HRSTEM images of QWs with quantitative analysis of composition and strain. (**a,b**) Cross-sectional HAADF-HRSTEM images recorded along [${\overline{1}}{\overline{1}}20$] from samples A1 and A3, respectively. (**c,d**) Corresponding nanoscale maps showing the variation of the *c*-lattice parameter. (**e,f**) Corresponding composition profiles extracted from the quantification of the Z-contrast. The image-simulated profiles for the indium contents of 33% and 50% are also overlapped in **(e,f)** respectively.

The lattice strain ${\varepsilon }_{z}^{l}$ along [0001] i.e., the reduced relative variation of the *c-*lattice constant in the MQW heterostructures, was determined from the experimental HR(S)TEM images using peak finding to determine the atomic column positions. Figure 3 illustrates representative HAADF-HRSTEM images with maps of the *c*-lattice parameter from two samples in the A-series. On such maps, the strain was averaged for ≈10–15 nm along the in-plane [1${\overline{1}}00$] direction, and the value per (0002) ML was then extracted. Up to 20 images were measured for each sample, and averages with standard deviations were calculated in order to consider any inhomogeneities.

**Figure 4:**
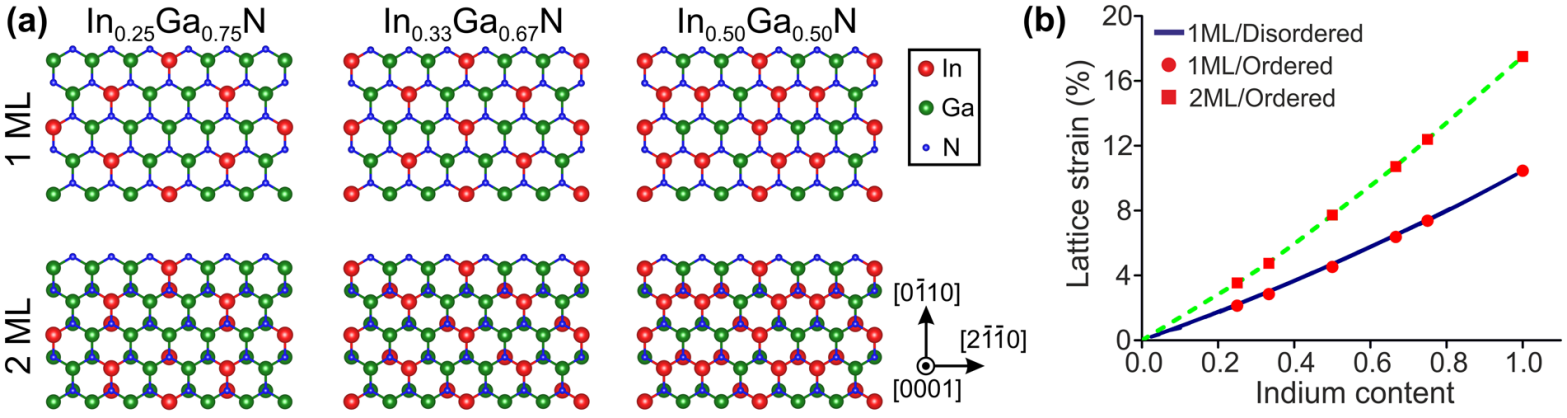
(**a**) Schematic illustrations in plan-view (i.e., along [0001]) of the ordered 1 ML and 2 ML QW structures up to the indium content of *x*_In_ = 50%. (The higher compositions of *x*_In_ = 67% and 75% can be obtained by the interchange of indium with gallium atoms.) (**b**) Lattice strain along [0001], ${\varepsilon }_{z}^{l}$, with respect to the indium content of the QW, *x*_In_, shown for the 1 ML ordered and disordered atomic distributions, as well as for the 2 ML ordered.

In order to correlate the strain measurements to the indium content, the strain-composition coupling in the ultra-thin In_*x*_Ga_1−*x*_N/GaN QWs was determined theoretically using atomistic calculations with the ternary MEAM (modified embedded atom method) interatomic potential^32^. A series of pseudomorphic supercells were relaxed, comprising 1 or 2 ML QWs. Initially we considered ordered QWs which, despite not being experimentally observed in our samples, are low energy structures. Then the influence of alloy disorder was investigated for supercells with 1 ML QWs generated with a compositional step of 5% and 100 random configurations for each step i.e., 2,000 supercells overall. Figure 4a illustrates the ordered structures that were considered. The results of the atomistic calculations are summarized in Fig. 4b, whereby it is seen that the strain of 1 ML ordered and disordered QWs is similar. Moreover, it is much reduced compared to that of the 2 ML QWs. The latter are closer to the prediction of anisotropic elasticity.

**Figure 5:**
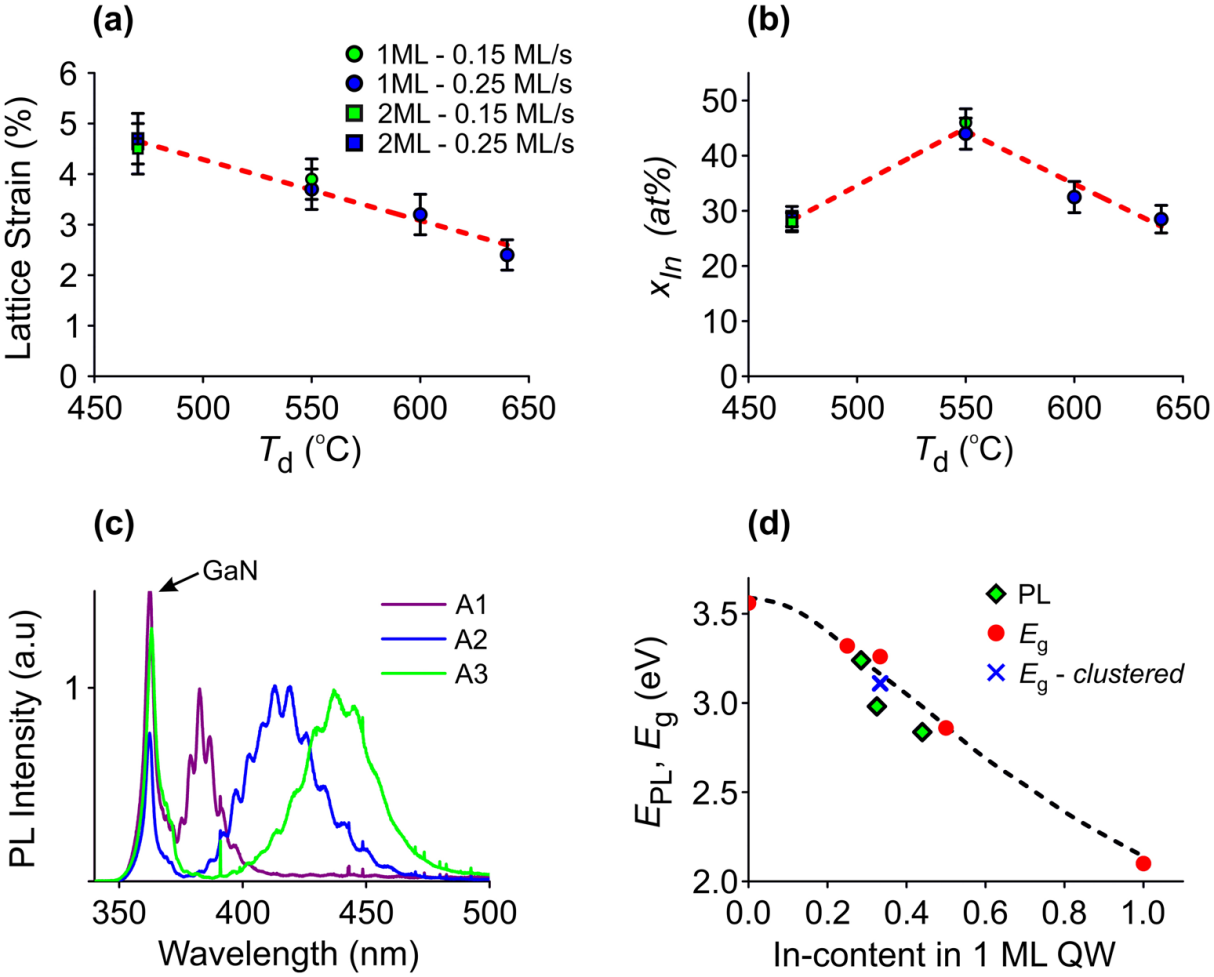
Collective results obtained from the quantitative HRSTEM image analysis and PL measurements. (**a**) Lattice strain, ${\varepsilon }_{z}^{l}$, with respect to the QW deposition temperature *T*_d_, showing linear trend. (**b**) Indium alloy content *x*_In_ with respect to *T*_d_. Trendlines are guides to the eye. (Labels in (**a**) and (**b**) indicate the observed QW thickness and the N*-limited growth rate.) (**c**) Normalized 300 K PL spectra from the samples of series A. (**d**) Peak PL energy of the samples of series A, as a function of the QW indium content (green symbols). The red symbols are theoretical predictions of energy gap extrapolated from the calculations of Ref.^7^, and the blue symbol is the theoretical extrapolation of 33% indium content with indium clustering.

Figure 5a shows the collective results of experimental strain measurements from all samples, and Table 1 lists the resulting QW indium contents, as extracted from the strain using the theoretically determined strain-composition coupling of Fig. 4b. In Fig. 5a, it is seen that ${\varepsilon }_{z}^{l}$ increases almost linearly with decreasing *T*_d_. The highest strain values (> 4.5%) were obtained for *T*_d_ = 470 °C which, however, yields 2 ML QWs on average. For the 1 ML QWs, the highest lattice strain was 3.9% at 550 °C. Slowing down the growth rate and doubling the In/N* flux ratio did not lead to an increase in strain that would indicate higher indium incorporation. From the results of Table 1 it is seen that the indium content appears to exceed 40% for samples A3 and A4. However, although strain provides an efficient and fast way to determine the composition, this is based on the assumption of fully pseudomorphic and perfectly flat QWs. Therefore, it is also necessary to ascertain the QW composition directly i.e*.*, through quantification of the Z-contrast on HAADF-HRSTEM images, which is far more demanding computationally. This task is undertaken in the next section.

### Z-contrast & PL analysis

The average chemical composition of the QWs was determined using cross-sectional observations along [${\overline{1}}{\overline{1}}20$] by comparing the experimentally measured intensity ratios of atomic columns, *I*_QW_/*I*_GaN_, in HAADF-HRSTEM images to simulated ones, for the same foil thickness. For this purpose, a series of simulated images was generated with the multislice approach under the “frozen phonon” approximation^33,34^, modelling the interaction of the electron beam’s wave function with the sample. The aforementioned energetically relaxed In_*x*_Ga_1−*x*_N/GaN supercells were employed as input for the image simulations in order to include the strain and static atomic displacements (SADs). Atomic column intensity measurements on experimental and simulated HAADF-HRSTEM images were obtained after peak finding, using image tessellation into Voronoi polygons^35^. The intensity of each polygon is related to the thickness of the atomic column, its indium content, and the influence of neighboring columns. Maps of the simulated *I*_QW_/*I*_GaN_ across the composition and foil thickness ranges were constructed (see Supplementary Fig. S4 online). By averaging the experimental intensity ratios over multiple Voronoi cells along the in-plane [1${\overline{1}}$00] direction, the average indium content of each ML was obtained, permitting the construction of concentration profiles along the [0001] growth direction. The error of the composition determination was obtained from the intensity fluctuations in the GaN barriers.

Figure 3e,f illustrate representative graphs of the indium content extrapolated from HAADF-HRSTEM intensity measurements of samples A1 and A3, respectively. The intensity enhancement of MLs abutting to the QW was attributed to the cross scattering, based on the multislice simulations, leading to an artificial indium content for them. Overall, better agreement with the strain measurements was obtained when indium was considered disordered on the QW, as ordering tends to decrease the HAADF-HRSTEM intensity^36^. This confirms further the disordered status of our QWs. The determined indium contents based on Z-contrast quantification are listed in Table 1 for all samples, verifying the high compositions (close to 50%) in samples A3 and A4, grown at 550 °C. The graph of Fig. 5b shows indium contents (as averages from the Z-contrast and strain measurements) with respect to *T*_d_. The 2 ML QWs contain less indium despite their higher strain^25^.

Figure 5c illustrates PL spectra from the 1 ML QWs of sample series A. The peak emission from the QWs shifts to longer wavelengths with decreasing *T*_d_, due to the increasing indium content. Ιn Fig. 5d, the PL peak energy is plotted as a function of the determined QW indium content, and our results are compared against theoretical predictions extrapolated for large barrier thicknesses from the DFT calculations of Gorczyca et al*.*^7^ The redshift of the experimental peak energies is due to the room temperature PL compared to the calculations being performed at 0 K. The relatively larger redshift of sample A2 (*T*_d_ = 600 °C) could be attributed to cation composition fluctuations that reduce the bandgap, and the corresponding theoretical prediction is included in Fig. 5d ^7^. Indeed, this sample exhibited local areas with indium content near 40%, as shown in Supplementary Fig. S5 online, and also had the highest PL intensity. The transition from low to high indium content appears to be continuous, probably mediated by the composition fluctuations i.e., regions of slightly increased indium concentration gradually coalesce along the ML. The PL results verify the pronounced increase of the indium content for *T*_d_ = 550 °C in agreement with the HRSTEM observations.

Overall, we have examined the influence of deposition temperature on the indium content of In_*x*_Ga_1−*x*_N/GaN MQWs under metal-rich conditions. After growth of each barrier, annealing and surface nitridation ensured consumption of any remaining adlayer. We have found that we can promote high quality 1 ML QW deposition down to *T*_d_ = 550 °C under this regime, with significantly increased indium content than previously reported, approaching 50%. Further decrease of the deposition temperature led to a structural deterioration of the barriers, making it necessary to apply an increase of the barrier growth temperature *T*_b_ after deposition of the first 1 nm of barrier thickness. Below 500 °C, we observed indium enrichment of a second ML, leading to corrugated 2 ML QWs with indium content close to 30%.

## Discussion

In the growth cycle of an In_*x*_Ga_1−*x*_N/GaN MQW, the deposition of InN follows the high temperature annealing, nitridation, and growth interruption (GI) stages (see Fig. 1). These stages dry the surface out of any metal adlayer/s or adatoms. Under these conditions i.e*.*, with indium shutter open and gallium shutter closed, and considering that the decomposition of GaN is kinetically hindered, only InN growth can be considered. It has been proposed that a single ML of InN on GaN can be stabilized at considerably higher temperatures than fully relaxed thick InN epilayers, due to a host matrix strengthening effect^17^. In order for InN to be thermodynamically stable against decomposition, its chemical potential should be lower than the sum of the indium and nitrogen chemical potentials in the liquid (for the indium species only) and/or the gas phases. Considering that relaxed (0001) InN films are unstable above *T*_dec_ ≈ 500 ^o^C^37,38^, the host matrix strengthening effect can be assessed by calculating the chemical potential of *n* MLs of pseudomorphic InN on GaN with respect to the chemical potential of fully relaxed thick InN epilayers, $\Delta \mu$. The former is calculated as $\left({E}_{n}^{\mathrm{tot}}-{E}_{\mathrm{GaN}}^{\mathrm{tot}}\right)/n$, where ${E}_{n}^{\mathrm{tot}}$ and ${E}_{\mathrm{GaN}}^{\mathrm{tot}}$ are the total energies per 1 × 1 surface unit cells of the slabs with and without *n* MLs InN, respectively. Furthermore, we consider three different growth conditions i.e., N-rich, moderate In-rich (single indium adlayer), and In-rich (indium bilayer i.e., double adlayer).

**Figure 6:**
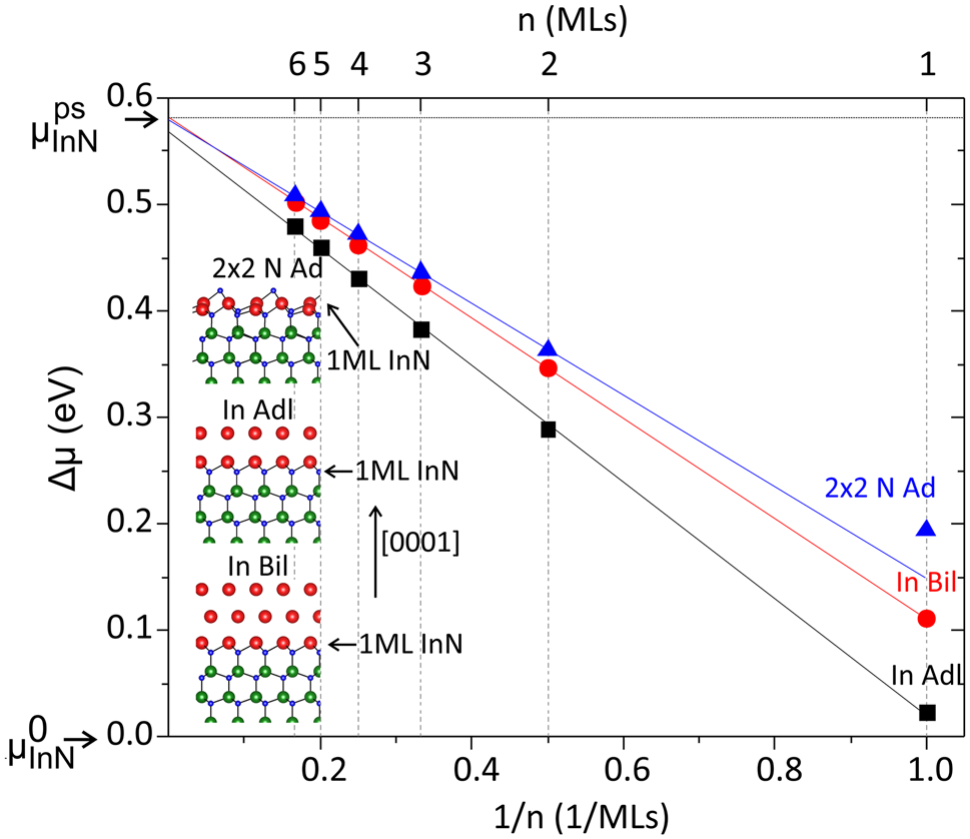
Chemical potential of InN epilayers on (0001) GaN with respect to the chemical potential of relaxed InN, $\Delta \mu ,$ as a function of the number of MLs, *n*. Three different surface terminations/growth conditions are considered: 2 × 2 N adatom reconstruction under N-rich conditions (2 × 2 N Ad), indium adlayer under moderate In-rich conditions (In Adl), and indium bilayer under In-rich conditions (In Bil). The chemical potentials of relaxed InN and InN biaxially strained to GaN are denoted as ${\upmu }_{InN}^{0}$ and ${\mu }_{\mathrm{ InN}}^{\mathrm{ps}}$, respectively. The solid lines are linear fits to data of the four thicker InN layers. Inset: Cross sectional schematic representations of the respective surface morphologies. The coloring of atoms in the ball-and-stick models is as in Fig. 4.

The results are shown in Fig. 6. As can be seen, the chemical potential of InN increases with epilayer thickness and tends to the chemical potential of pseudomorphic thick InN (${\mu }_{\mathrm{ InN}}^{ps}$) for larger thicknesses. This indicates that the first InN ML is indeed strongly bound to the GaN surface and is more stable at higher temperatures compared to thick InN epilayers. This strengthening effect arises from the fact that Ga-N bonds are stronger than In-N bonds (0.39 eV/bond difference in cohesive energies)^39,40^: at the (0001) surface, the nitrogen atoms of the InN ML form three bonds with indium atoms and one bond with a gallium atom. In contrast, nitrogen atoms in InN form four bonds to indium atoms. However, despite this strengthening effect, Fig. 6 shows that the chemical potential of a single InN ML on GaN is still larger than that of relaxed InN (${\mu }_{\mathrm{ InN}}^{0}$). Hence, the single ML of InN on GaN still decomposes at lower temperature than relaxed InN, and the host matrix strengthening cannot support the stabilization of a binary InN ML on GaN at temperatures above ≈500 °C. Therefore, our experimental observations are not interpreted within the context of the sheet-island-like QW model comprising binary InN as proposed by Yoshikawa et al.^19^.

**Figure 7:**
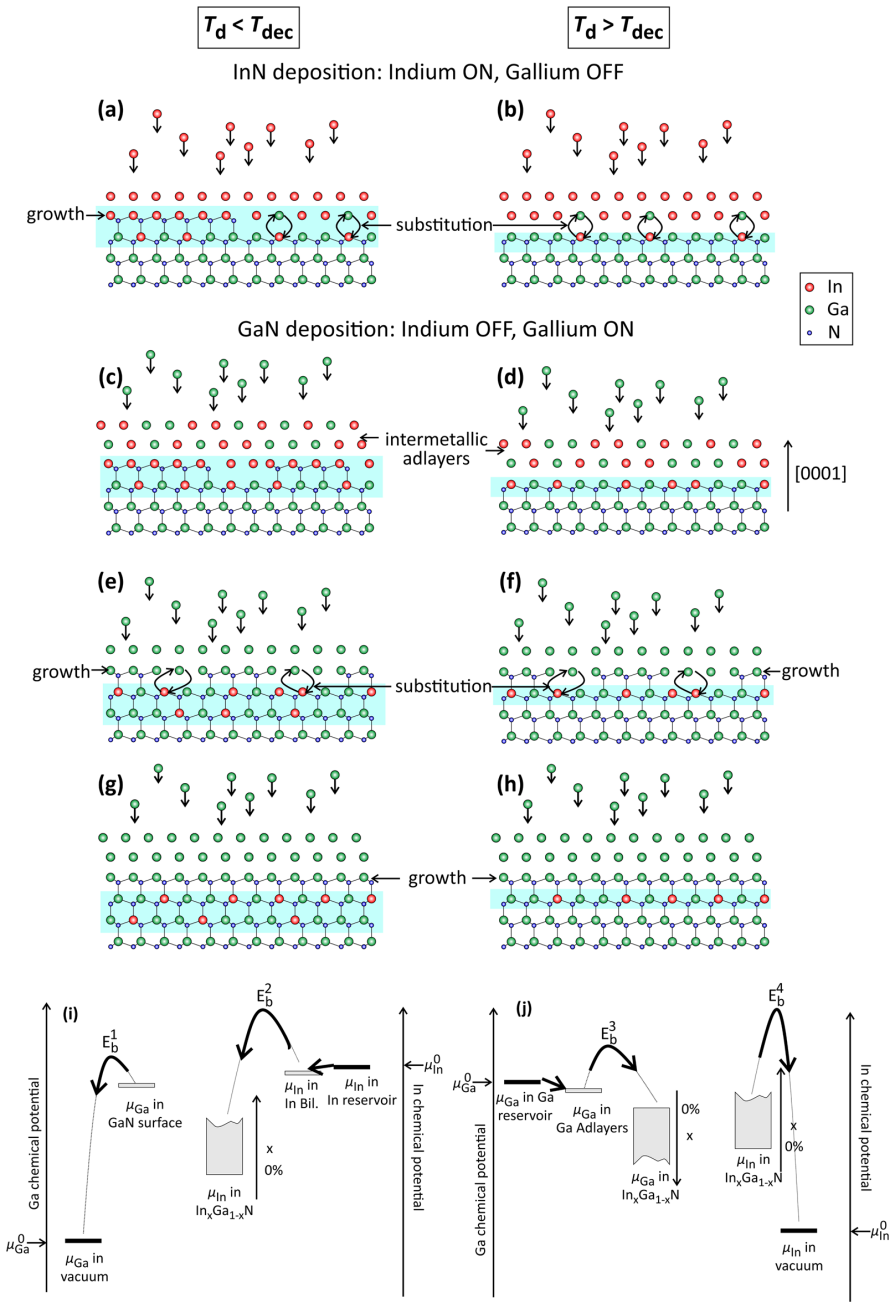
(**a–h**) Schematic representation of the synthesis of In_*x*_Ga_1−*x*_N/GaN MQWs. In the left (right) figures, the deposition temperature is lower (higher) than the decomposition temperature, respectively. (**a,b**) InN deposition stage. (**c–h**) GaN deposition stage. The ball-and-stick models are in cross-sectional view and the color code is as in Fig. 4. The blue-shaded areas indicate the position of the QW. (**i,j**) Schematic representation of gallium and indium chemical potentials in the InN and GaN deposition stages, respectively. The black bars indicate the boundaries of indium and gallium chemical potentials imposed by the growth conditions, ${\mu }_{\mathrm{In}}^{0}$ and ${\mu }_{\mathrm{Ga}}^{0}$, respectively. Under thermodynamic equilibrium, ${\mu }_{\mathrm{Ga}}$ and ${\mu }_{\mathrm{In}}$ at the surface or in In_*x*_Ga_1−*x*_N become equal to ${\mu }_{\mathrm{Ga}}^{0}$ and ${\mu }_{\mathrm{In}}^{0}$, respectively. ${\mu }_{\mathrm{Ga}}$ and ${\mu }_{\mathrm{In}}$ in In_*x*_Ga_1−*x*_N depend on the indium content *x*: ${\mu }_{\mathrm{In}}$ (${\mu }_{\mathrm{Ga}}$) increases (decreases) with *x*, respectively. ${E}_{b}^{i}$ ($i$ =1…4) denote kinetic barriers for indium and gallium atoms to move from the metastable to the stable phases.

In order to shed light on the deposition of these In_*x*_Ga_1−*x*_N QWs, the changes in the gallium and indium chemical potentials during the different stages of the growth cycle (given in Fig. 1) are examined. As mentioned already, the InN deposition stage starts with a dry surface. During InN deposition, the Ga shutter is closed and ${\mu }_{\mathrm{Ga}}$ is fixed to an arbitrary low value as shown in Fig. 7i. On the other hand, the indium shutter is open and ${\mu }_{\mathrm{In}}$ is fixed to the chemical potential of the indium source, leading to formation of a single or double indium adlayer which is stable up to ≈650 °C (see Fig. 7a,b)^41,42^. If *T*_d_ is lower than the InN decomposition temperature i.e., $\lesssim$ 500 °C (sample series B), InN growth is favorable. The excess indium remains in adlayer form. However, as discussed earlier, above *T*_dec_ (sample series A) the growth of InN is thermodynamically unfavorable, while the indium adlayer is still stable on GaN. In the GaN deposition stage, the reverse situation occurs i.e., ${\mu }_{\mathrm{In}}$ is fixed to an arbitrary low value (indium shutter closed), ${\mu }_{\mathrm{Ga}}$ is fixed to the chemical potential of the gallium source, as shown in Fig. 7j, and growth of the GaN barrier takes place.

Therefore, growth of InN is thermodynamically allowed only for sample series B. This is in apparent contrast to the experimental observation of 1 ML QWs at *T*_d_ well above *T*_dec_ in series A. It has been proposed recently that 1 or 2 ML In_*x*_Ga_1−*x*_N QWs can be kinetically stabilized at the onset of the GaN deposition stage^24^. Indeed, at the end of indium deposition, the surface is covered by a single or double indium adlayer. In the succeeding stage, the supplied gallium would tend to diffuse under this adlayer and preferentially occupy sites on the GaN surface^43^. Then, for a short finite period of time, an In-Ga intermetallic of thickness at least equal to the thickness of the adlayer is present on the surface, as illustrated schematically in Fig. 7c,d. If *T*_b_ is below the decomposition temperature of In_x_Ga_1−x_N, an In_x_Ga_1−x_N QW could be grown from the intermetallic and be kinetically stabilized.

However, the mechanism of In_x_Ga_1−x_N growth from an intermetallic adlayer is in contrast to our observations: in both sample series A and B, the amount of indium present in the adlayer under metal-rich conditions at the beginning of the GaN deposition stage is at least 2 MLs. Furthermore, excess indium inside surface droplets is sufficiently mobile at 610 °C to wet the surface, providing an extra indium reservoir^44^. Therefore, it would be expected that growth from the In-Ga intermetallic adlayer would result in QWs thicker than 1–2 MLs and/or with a diffuse upper interface. These contradict our findings of QWs with maximum 2 MLs thickness and rather abrupt interfaces. Moreover, growth from an In-Ga intermetallic is in contrast to recent reports on nitrogen-rich realization of In_x_Ga_1−x_N/GaN MQWs^21,23,45^, whereby it was reported that such In_*x*_Ga_1−*x*_N QWs are restricted to *x*_In_ = 25% and 1 ML thickness accompanied by a characteristic × 3 RHEED (reflection high-energy electron diffraction) pattern. Although the single ML thickness and its indium content could be attributed to In_x_Ga_1−x_N growth from a 2 × 2 indium adatom reconstruction^46^ i.e., 0.25 ML indium adatom surface content at the beginning of the GaN deposition stage, this is in contrast to the observed × 3 RHEED pattern. The latter is explained in terms of an ordered $\left(2\sqrt{3}\times 2\sqrt{3}\right)\mathrm{R}30^\circ$ In_*x*_Ga_1−*x*_N configuration i.e., indium has already been incorporated in the topmost GaN surface layer at the end of the InN deposition, although no gallium is supplied at this stage.

Based on the above discussion, we propose a substitutional synthesis model of sub-nm thick In_x_Ga_1−x_N QWs. As can be seen in Fig. 7i,j, there is a strong driving force for indium (gallium) to substitute for gallium (indium) in the first (second) stage of the growth cycle. In sample series B i.e., for *T*_d_ < *T*_dec_, two mechanisms are active during the InN deposition stage: (i) indium substitutes for gallium, and (ii) growth of InN takes place (see Fig. 7a). This results in 2 MLs, with the first one being In_x_Ga_1−x_N and the second InN. In sample series A, where *T*_d_ > *T*_dec_, a single In_x_Ga_1−x_N ML is obtained at the end of the InN deposition stage only through the substitutional mechanism (see Fig. 7b). The maximum indium content is limited by the *T*_dec_ of In_x_Ga_1−x_N and the actual indium content is defined by kinetics i.e., the substitutional kinetic barrier, the temperature, and the time the surface is exposed to indium flux and/or indium adlayer(s). The latter corresponds to the duration of the InN deposition stage i.e., in the order of 3–4 s.

In the stage of GaN deposition, the two active mechanisms are: (i) gallium substituting for indium and (ii) growth of GaN (see Fig. 7e,f). As in the first stage, substitution is controlled by kinetics, but the relevant time scale is provided by the growth rate of GaN which is ≈1 s (the time required to grow 1 ML of GaN barrier). The substitutional mechanism in the second stage reduces the indium content in the QW but, thanks to the smaller time scale, a considerable amount of incorporated indium is kinetically stabilized. In sample series B, the second ML of the QW is more affected than the first by the substitution and transforms from to InN to In_x_Ga_1−x_N. After formation of the first complete GaN ML above the QW, barrier growth proceeds normally from the gallium adlayer as shown in Fig. 7g,h.

In summary, when *T*_d_ < *T*_dec_*, *i.e*.*, in sample series B, both the InN growth and substitutional mechanisms are active, while when *T*_d_ > *T*_dec_, i.e., in sample series A, In_*x*_Ga_1−*x*_N is synthesized only via substitution. Overall, the substitutional synthesis of In_*x*_Ga_1−*x*_N QWs is a complex interplay between thermodynamics and kinetics at the InN and GaN deposition stages: on the one hand, higher temperatures and/or longer exposure of the GaN surfaces to indium fluxes and/or indium adlayer(s) enhance indium substitution at the InN deposition stage. On the other hand, higher temperatures limit the maximum indium content due to decomposition and enhance gallium substitution at the GaN deposition stage. Reduced growth temperatures at the GaN deposition stage result in microstructural deterioration of the barrier. The kinetic barriers ${E}_{b}^{i}$ (*i* = 1,..,4) for substitution and desorption (see Fig. 7i,j) are dominant microscopic parameters that control the indium content. Although, the calculation of these barriers is beyond the scope of the present work, the aforementioned discussion indicates that the dependence of the indium content on temperature is not monotonous. Nevertheless, our model provides important and significant insights on the synthesis of sub-nanometer thick In_*x*_Ga_1−*x*_N QWs and elucidates the key parameters that should be employed to maximize indium content and optimize the structural quality of the MQWs.

## Conclusions

Using PAMBE under highly metal-rich growth conditions, we have deposited In_*x*_Ga_1−*x*_N/GaN MQWs aiming to increase their indium content and elucidate the elusive growth mechanisms of QWs with sub-nanometer thickness. Such QWs can be deposited by exploiting the 1–2 ML-thick In/Ga metallic adlayer in order to kinetically stabilize quasi-2D In_*x*_Ga_1−*x*_N QWs among GaN barriers. To this end, we have studied QW deposition temperatures both above and below the InN decomposition limit. In the latter case, the growth temperature of the barriers was ramped up to maintain a high crystal quality with no extended defects. An integrated framework of advanced HR(S)TEM methodologies permitted the quantitative determination of the indium content in these QWs either directly, through the atomic-scale quantification of Z-contrast mediated by image simulations, or indirectly by strain measurements with sub-nanometer spatial resolution. Both approaches necessitated a comparison of the experimental observations to energetically relaxed atomistic supercells. Our analysis has shown that record-high indium contents of about 45% can be achieved in 1 ML-thick quasi-2D QWs grown above the InN decomposition limit. Below this limit, 2 ML QWs were obtained albeit with lower indium content. Since binary InN stabilization is thermodynamically forbidden above the decomposition limit, we have proposed a growth model based on substitutional synthesis, taking place through the formation of an In/Ga metallic adlayer. The model appears to account for both the 1 ML and 2 ML QW deposition by considering the pertinent atomic scale processes. The achieved indium content in these QWs surpasses by far the previously perceived compositional limits, and could be further increased in the future, thus making it promising to enable topological insulator applications integrated to the compound semiconductor technology, as well as advanced bandgap engineering for ultra-efficient chromatically tunable III-nitride optoelectronic devices.

## Methods

### PAMBE growth & photoluminescence spectroscopy

InN/GaN MQWs were grown by PAMBE with 10 nm GaN barriers on metal-polarity (0001) GaN/Al_2_O_3_ templates. A 200 to 400 nm GaN buffer was first deposited. The QWs were grown under metal-rich conditions with In:N* flux ratios of 3:1 or 6:1, and N-limited InN growth rates of 0.25 or 0.15 ML/s. The nominal QW thickness was 1 ML. Slightly Ga-rich conditions were employed during barrier growth to prevent indium incorporation. At the end of each barrier, a 2 min anneal at 685 °C, followed by 1 min exposure to active nitrogen at the same temperature, were employed to thoroughly consume remaining Ga adatoms prior to deposition of the next QW.

PL measurements were performed at 300 K on samples comprising 50 nm GaN capping layers, using excitation by a cw He-Cd laser at 325 nm. The PL signal from the sample was measured by a monochromator and a very sensitive LN2 cooled CCD system.

### HR(S)TEM observations & strain measurements

Cross-sectional HRTEM/HRSTEM observations were performed along the [${\overline{1}}{\overline{1}}20$] and [1${\overline{1}}00$] zone axes. Sample were prepared by wedge polishing followed by Ar^+^ ion milling in the Gatan PIPS starting at 5 kV and finishing at 0.8 kV. HRTEM observations were performed using a 300 kV image aberration-corrected Thermo Fisher Scientific (FEI) Titan Themis 60/300 instrument and a conventional 200 kV JEOL JEM 2011 microscope. HRSTEM observations were performed in a 300 kV probe-corrected Thermo Fisher Scientific (FEI) Titan Themis 60/300 microscope. HAADF images were obtained from foil regions with thicknesses between 25 and 90 nm using a semi-convergence angle of 23.8 mrad and detector semi-collection angles of 78–200 mrad, setting the fast scan direction normal to the *c*-plane. The dwell time was 1 μs px^−1^ to eliminate any beam-induced indium clustering. Averaging of 30 frames was conducted accounting for sample drift and scan noise.

For strain measurements, peak finding was performed on HRSTEM images using the Atomap software^47^ to determine atomic column positions by identifying intensity maxima using 2D Gaussian fitting. A peak-pairs analysis algorithm was employed utilizing two interpenetrating sublattices, consistent with the hexagonal …*ABAB*… stacking. Then the reduced relative variation of the *c*-lattice parameter relative to that of GaN was extracted. In addition to peak finding, geometrical phase analysis (GPA)^48^ was also employed, using the *g*0002 spatial frequency with a 0.5 nm spatial resolution, for fast strain determination from HR(S)TEM images.

### Multislice simulations

HAADF-HRSTEM image intensity was quantified at atomic resolution using multislice image simulations performed along the [${\overline{1}}{\overline{1}}20$] zone axis with the STEMsalabim software^49^. The simulations utilized relaxed atomistic supercells obtained by the empirical potential calculations to include the strain and SADs^50^. The supercell size was 12[1${\overline{1}}00$] × 17[0001] whereas the foil thickness was varied to evaluate the influence of beam spreading and cross-scattering on the intensity distribution of the QW and its abutting barrier MLs^25,51^. Thermal diffuse scattering (TDS) was considered using the “frozen phonon” approximation^33^. Ten phonon configurations with uncorrelated atomic thermal vibrations at 300 K were deemed sufficient to simulate accurately the image intensity^52,53^. Considering the aberration coefficients, the effective source size was obtained by convoluting with a 2D Gaussian of full width at half-maximum FWHM = 0.04 nm to allow agreement between experimental and simulated intensities of GaN atomic columns at the same foil thickness^54^. The sensitivity and geometry of the annular dark field detector were considered by acquiring detector scans to normalize image intensities^55^. The influence of amorphous layers due to the ion milling was considered in the manner detailed elsewhere^25^. Also, in order to identify any influence of the thin foil relaxation^35,56^, additional multislice simulations were performed on supercells comprising relaxed free surfaces normal to the projection direction and it was found to be negligible for the employed foil thicknesses.

### Empirical potential calculations

Atomistic calculations were performed utilizing a ternary MEAM potential^32^ in the classical molecular dynamics code LAMMPS^57^. The reliability of the potential for In_x_Ga_1−x_N has been confirmed through comparison with DFT calculations^58^. We considered both ordered and disordered indium arrangements in the QWs. For disordered QWs, orthorhombic supercells with size 16*a*_GaN_ along [${\overline{1}}{\overline{1}}20$], 9*a*_GaN_ $\sqrt{3}$ along [1${\overline{1}}00$], and 8*c*_GaN_ along [0001] (where *a*_GaN_ and *c*_GaN_ are the GaN lattice parameters), comprising overall 9216 atoms, were relaxed using periodic boundary conditions whereby the QW was constrained to remain pseudomorphic. For the ordered QWs, we considered orthorhombic periodic supercells sized to 6*a*_GaN_ along [${\overline{1}}{\overline{1}}20$], 2*a*_GaN_ $\sqrt{3}$ along [1 ${\overline{1}}$ 00], and 17*c*_GaN_ along [0001], comprising 1632 atoms.

### Density functional theory calculations

DFT calculations were performed within the local-density approximation (LDA) and the projector augmented-wave (PAW) method^59,60^. The indium and gallium 3*d* electrons were treated as valence states. The plane-wave energy cutoff was 450 eV and an equivalent of an 8 × 8 × 1 Monkhorst–Pack *k*-point mesh for the 1 × 1 surface unit cell was used to sample the Brillouin zone (BZ). The surfaces were modeled using a slab geometry consisting of 8 metal-nitrogen monolayers separated by a vacuum region of 25 Å and the 1 × 1 and 2 × 2 surface unit cells for the metal-rich and N adatom reconstructions, respectively. Nitrogen atoms at the bottom side of the slab i.e., the $\left(000{\overline{1}}\right)$ surface, were passivated with pseudo-hydrogen atoms of partial charge 0.75.

## Supplementary Information

Supplementary Figures.

## Data Availability

The datasets generated during and/or analyzed during the current study are available from the corresponding author on reasonable request.
