# Supplementary material for: Substitutional synthesis of sub-nanometer InGaN/GaN quantum wells with high indium content
Source: Sci Rep. 2021 Oct 18;11:20606. doi: 10.1038/s41598-021-99989-0 (PMC8523525; doi:10.1038/s41598-021-99989-0)
Supplement: Supplementary file 1 — Supplementary Figures. [file 41598_2021_99989_MOESM1_ESM.pdf]

Substitutional synthesis of sub-nanometer InGaN/GaN quantum wells with high indium content

I. G. Vasileiadis[1], L. Lymperakis[2], A. Adikimenakis[3], A. Gkotinakos[1], V. Devulapalli[2], C. H. Liebscher[2], M. Androulidaki[3,4], R. Hübner[5], Th. Karakostas[1], A. Georgakilas[3,4], Ph. Komninou[1], E. Dimakis[5], and G. P. Dimitrakopulos[1*]

[1]Department of Physics, Aristotle University of Thessaloniki, Thessaloniki, Greece

[2]Max-Planck Institut für Eisenforschung GmbH, Düsseldorf, Germany

[3]Microelectronics Research Group (MRG), IESL, FORTH, Heraklion, Greece

[4]Department of Physics, University of Crete, Heraklion, Greece

[5]Institute of Ion Beam Physics & Materials Research, Helmholtz-Zentrum Dresden-Rossendorf, Dresden, Germany

* email: gdim@auth.gr

Supplementary Information

- **Figure S1:** Representative cross-sectional HRTEM images of the MQW heterostructures and respective maps of the lattice strain along the [0001] direction, obtained by GPA.
- **Figure S2:** Cross-sectional bright field TEM images of MQW heterostructures deposited at 470 °C with and without the temperature ramping in the barriers. The images serve to illustrate the defect-free MQW heterostructure when the barrier growth temperature is increased to T_b = 550 °C.
- **Figure S3:** HRSTEM image and strain map of the first two QWs in the MQW heterostructure deposited at 470 °C without the temperature ramping in the barriers, to show that, in sample series B, the QW composition and thickness were not significantly affected by the increased T_b, while the overall crystal structure of the MQW improved dramatically through the elimination of stacking faults and cubic pockets.
- **Figure S4:** (**a**) Representative multislice image simulations, illustrating also the image tessellation into Voronoi polygons. (**b**) Maps of the calculated I_{QW}/I_{GaN} intensity ratio with respect to the indium content and TEM foil thickness.

- **Figure S5:** HRTEM images showing two QW regions in sample A2 with superimposed maps of the lattice strain. In one region the lattice strain is rather homogeneous and in the other there are nanometer-sized regions of higher strain.

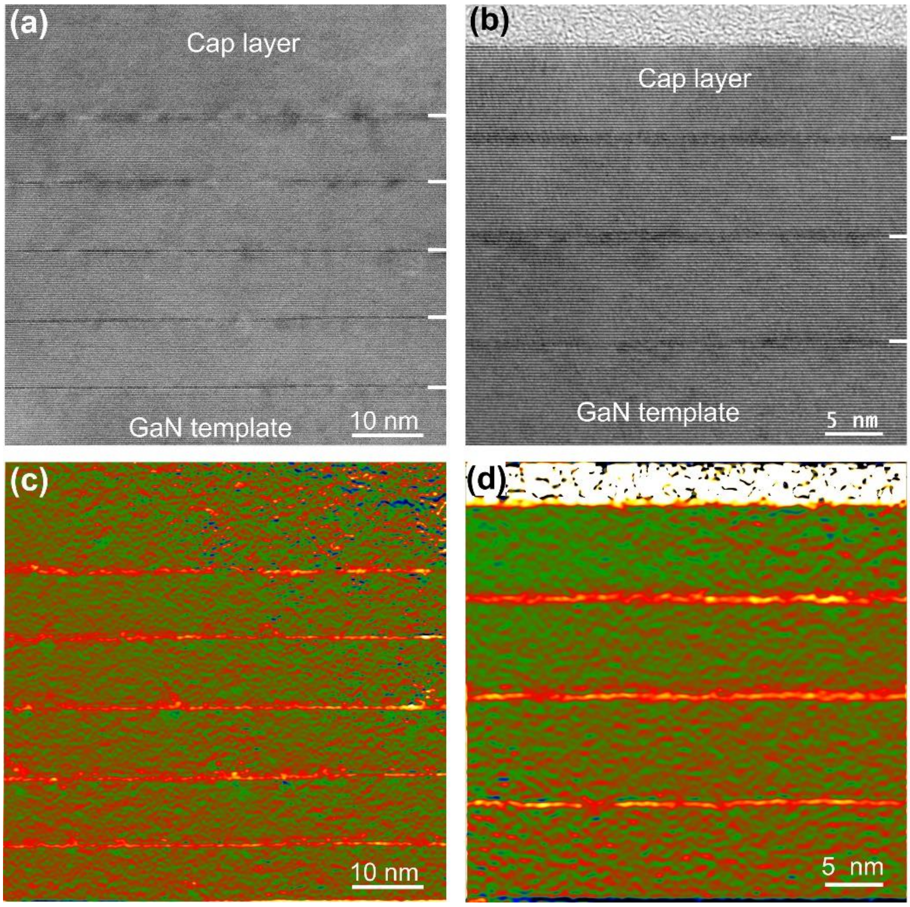

Supplementary Figure S1. Representative cross-sectional HRTEM images of samples (**a**) A3 and (**b**) B3 illustrating the whole MQW heterostructures of 5 and 3 QWs respectively. The images were obtained off the [$\overline{11}$20] zone axis with a *g*±0002 Laue condition to keep only the (0002) lattice fringes. The respective GPA maps of the lattice strain along the [0001] direction, illustrated in (**c**) for sample A3 and (**d**) for sample B3 serve to illustrate the distribution of the strain along the MQW heterostructures.

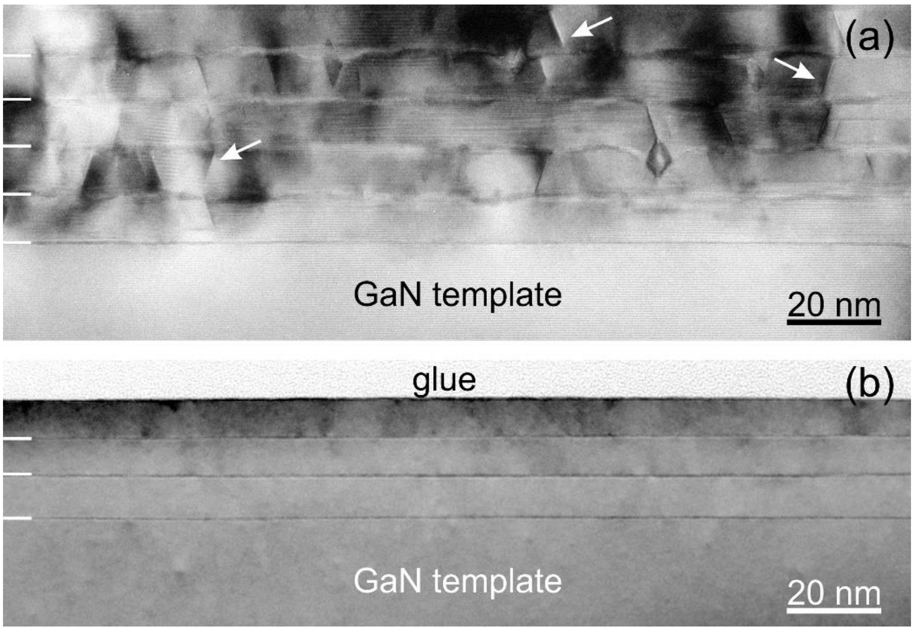

Supplementary Figure S2. (**a**) Cross-sectional bright field TEM image obtained near the $[\bar{1}\bar{1}20]$ zone axis with *g*0002, showing a MQW heterostructure comprising five QWs grown at 470 ^{o}C. The MQW exhibits a defected structure due to multiple stacking faults and regions of zincblende cubic structure. Arrows indicate {111} twin boundaries. White lines indicate the QW positions. Only the first QW of the MQW is well-formed. (**b**) Similar cross-sectional TEM image of sample B3 showing the defect-free heterostructure comprising three QWs deposited at T_d = 470 ^{o}C while the barrier growth temperature was increased to T_b = 550 ^{o}C.

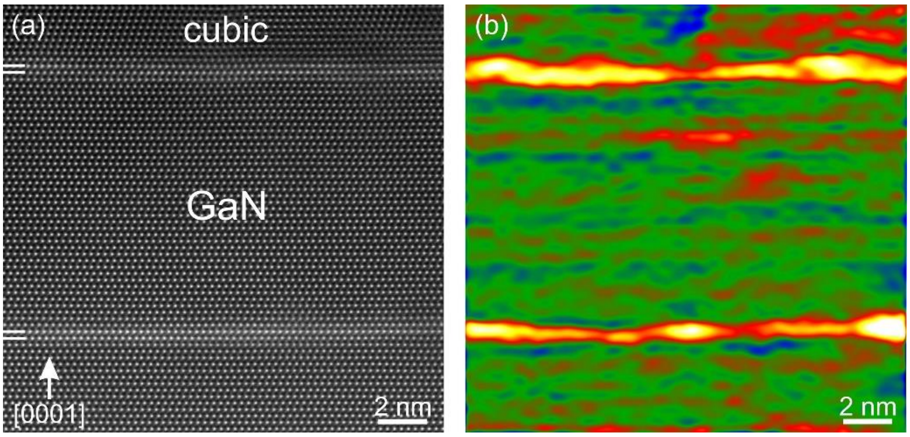

Supplementary Figure S3. (**a**) HRSTEM image of the first and second QW in the MQW heterostructure of Fig. S2(a) grown at 470 °C. Cubic GaN is indicated in the barrier over the 2nd QW. It is seen that at least the first QW clearly comprises 2 MLs and is similar to those of Fig. 2(d) in the paper. (**b**) Corresponding GPA map of the lattice strain along [0001]. The average strain measurement for the first QW was found equal to 4.4 ± 1.1% which is similar to the strain in the QWs of sample series B, showing that the QW composition and thickness in sample series B were not significantly affected by the increased growth temperature T_b of the GaN barriers whereas the crystal structure improved dramatically.

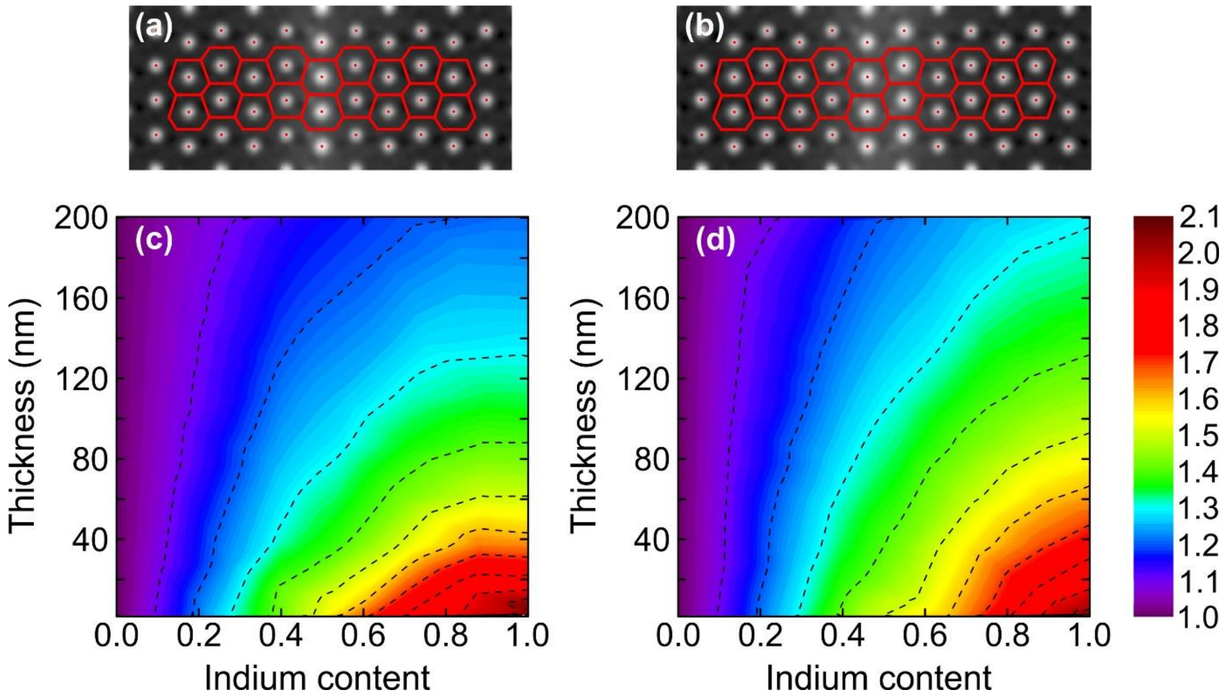

Supplementary Figure S4. (a) and **(b)** Simulated HRSTEM images of 1 ML and 2 ML QWs respectively, showing superimposed the peaks determined by peak finding and the tessellation into Voronoi polygons. The simulations are illustrated for $In_{0.67}Ga_{0.33}N$/GaN QWs at 49.7 nm sample thickness. **(c)** and **(d)** Maps of the calculated I_{QW}/I_{GaN} intensity ratio with respect to the indium content and TEM foil thickness for the 1 ML and the 2 ML QWs respectively under the pertinent imaging conditions.

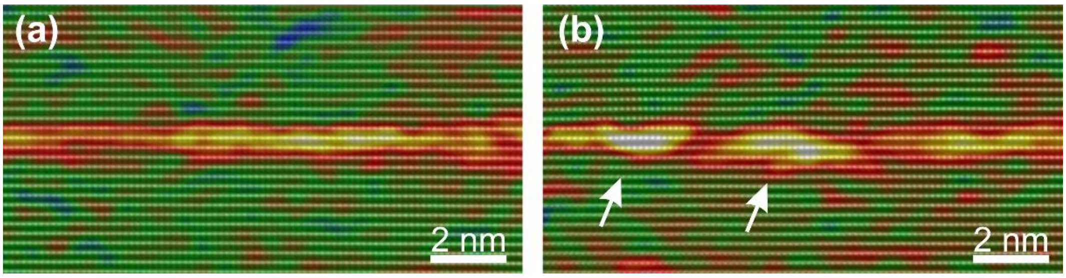

Supplementary Figure S5. HRTEM images along the $[1\overline{1}00]$ zone axis showing two QW regions in sample A2 with superimposed maps of the lattice strain along [0001] obtained by GPA. In (**a**) the lattice strain is rather homogeneous with an average value of ε_{zz}^{lt} = 3.1 ± 0.4 %. In (**b**) there are nanometer-sized regions of higher strain indicated by arrows. The average lattice strain is similar to (**a**), but the indicated local regions have lattice strain ≈ 4 %.
